# Recent progress in metal assisted multicomponent reactions in organic synthesis

**DOI:** 10.3389/fchem.2023.1217744

**Published:** 2023-09-08

**Authors:** Kokila Sakthivel, R. J. Gana, Toshitaka Shoji, Naoko Takenaga, Toshifumi Dohi, Fateh V. Singh

**Affiliations:** ^1^ Chemistry Division, School of Advanced Sciences, Vellore Institute of Technology (VIT), Chennai, Tamil Nadu, India; ^2^ Graduate School of Pharmaceutical Sciences, Ritsumeikan University, Kusatsu, Shiga, Japan; ^3^ Faculty of Pharmacy, Meijo University, Nagoya, Japan

**Keywords:** multicomponent reactions, arylation, cyclization, cycloaddition, metal, copper, palladium, catalyst

## Abstract

To prepare complicated organic molecules, straightforward, sustainable, and clean methodologies are urgently required. Thus, researchers are attempting to develop imaginative approaches. Metal-catalyzed multicomponent reactions (MCRs) offer optimal molecular diversity, high atomic efficiency, and energy savings in a single reaction step. These versatile protocols are often used to synthesize numerous natural compounds, heterocyclic molecules, and medications. Thus far, the majority of metal-catalyzed MCRs under investigation are based on metal catalysts such as copper and palladium; however, current research is focused on developing novel, environmentally friendly catalytic systems. In this regard, this study demonstrates the effectiveness of metal catalysts in MCRs. The aim of this study is to provide an overview of metal catalysts for safe application in MCRs.

## Graphical Abstract

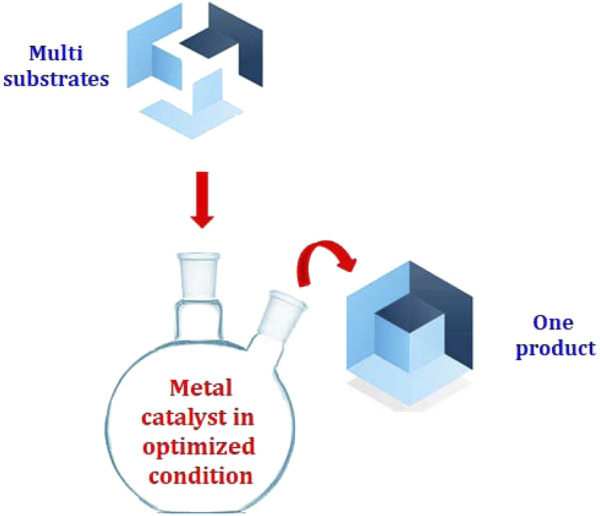

## 1 Introduction

The development of innovative and effective methods for the one-step synthesis of various complicated systems from basic, easily obtainable starting materials is a formidable objective in organic chemistry. Multicomponent reactions (MCRs) are chemical reactions wherein multiple initial materials combine to generate a target molecule; essentially, all or most of the initial material atoms contribute to the synthesized product (Ugi et al., 1994). Consequently, MCRs have encouraged synthetic chemists to focus on one-step synthesis of complicated organic compounds. Although MCRs have been used for more than a century, their utility is typically restricted to a small number of reactions because managing the appropriate selectivity and reactivity selectivity when using three or more components is challenging (Ugi and Steinbruckner, 1960; Bienayme and Bouzid, 1998; Groebcke et al., 1998; Kaim et al., 2005). MCRs exhibit better synthetic efficiency compared with normal multi-step reaction routes and offer other advantages, including rapid and easy operation, time and energy savings, high atom economy, and environmental friendliness (Biggs-Houck et al., 2010; Slobbe et al., 2012; Rotstein et al., 2014; Khan et al., 2015; Malinakova, 2015; Paprocki et al., 2018). MCRs are typically easy to execute experimentally and frequently without the need for dry conditions or an inert atmosphere. Using MCRs, molecules can be combined in a convergent rather than linear manner. Therefore, because all property-determining moieties are introduced simultaneously rather than sequentially, structure-activity relationships (SARs) can be quickly constructed using MCRs. In addition, more than 300 distinct scaffolds have been documented in chemical literature, and MCRs offer a tremendous amount of chemical variety (Domling and Ugi, 2000; Ganem, 2009; Domling and Huang, 2010; Domling et al., 2012; Brauch et al., 2013; Mostofi et al., 2018; Graebin et al., 2019). The regulation of selectivity is a crucial problem when various sets of reactants are used in these procedures. Nevertheless, this lack of selectivity enables the production of intricate adduct combinations that are useful in combinatorial chemistry for biological applications. However, the mechanisms behind how reactants spontaneously choose one another (innate selectivity) remain unclear, and this is possibly challenging to achieve under normal conditions. In this regard, numerous conditions can be considered, including structural, chemical, electrical, compositional, kinetic, and energetic considerations, which can affect selectivity (Ghashghaei et al., 2019). In homogeneous catalytic processes, some factors may be more significant than those in heterogeneous reactions, and *vice versa*. However, in most circumstances, these two important catalytic types do not offer significant distinctions, particularly for controlling product selectivity (Davis, and Suib, 1993). Transition-metal catalysts are currently being employed in multicomponent processes as catalytic platforms because they assist in the selectivity and reactivity of reactions. A single catalytic pathway can produce complex scaffolds from simple and easily accessible building blocks using metal catalysts to activate the starting components and control the selectivity of the reaction. These characteristics considerably influence the development of MCRs employing various metal catalysts (D’Souza and Muller, 2007; Sunderhaus and Martin, 2009; Cioc et al., 2014; Kakuchi, 2015; Haji, 2016).

## 2 Calcium

**FIGURE 1:**
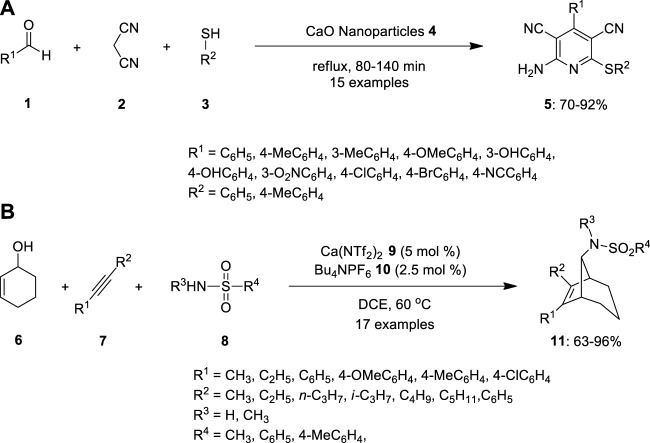
Synthesis of **(A)** substituted pyridines **5** and **(B)** bicyclic sulfonamides **11** using Ca-catalyst via multicomponent reaction (MCR).

Aryl substituted pyridines are affluented in medicinal chemistry, which have beed synthesized by the condensation of aldehydes **1**, thiols **3** and malononitrile **2** in the presence of calcium oxide nanoparticles **4** (Figure 1A). When compared to homogeneous base catalysts, calcium oxide (CaO) nanoparticles are inexpensive, have a high basicity, non-corrosive, economically benign, and are simple to handle. In terms of excellent yields, fast reaction times, reusability, and minimal catalyst loading, this method proffers an advanced method for the synthesis of 2-amino-4-aryl-3,5-dicyano-6-sulfanylpyridines **5**. When the reaction performed under the optimal conditions using a variety of aldehydes and thiols to evaluate the extent of this approach, the observed that in the beginning of the reaction, electron-withdrawing groups like NO_2_, Cl, and Br interacted quite easily with malononitrile, producing 2-amino-3,5-dicarbonitrile-6-thio-pyridines **4** in a short amount of time. Additionally, compared to unimpeded aldehydes, sterically hindered aldehydes reacted more slowly. When malononitrile, thiophenol, and benzaldehyde were used as substrates, the best results were obtained (Safaei et al., 2013).

Later, in place of pricey and extremely hazardous noble metal catalysts, Gao and team created a straightforward calcium catalyst that may be employed for a huge synthetic applications. The high Lewis acidity of the calcium catalyst efficiently transforms precursors into reactive carbocations, enabling reversible covalent bond formation due to its potency as a Lewis acid. The aliphatic alkynes as nucleophiles in carbocation cascade reactions to synthesize bicyclic sulfonamides (Figure 1B). The reaction proceeded through the ionization of hexenol **6** in the presence of Bu_4_NPF_6_ **10** and calcium(II) bis(trifluoromethanesulfonimide) 9. The team discovered that changing the substrates of the disubstituted sulfonamides had no effect. When a bulky group was introduced into the alkyne, the target product **11** was produced in excellent yield, along with complete diastereoselectivity (Gao et al., 2015).

## 3 Copper

**FIGURE 2:**
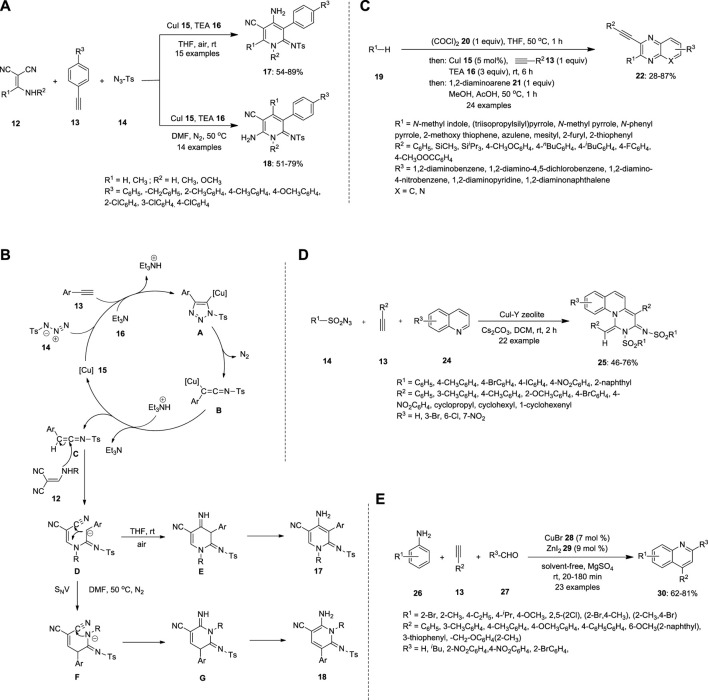
Synthesis of *N*-heterocyclic compounds **(A)** substituted pyridines **17** and **18**, **(B)** mechanism of pyridines **17** and **18**, **(C)** quinoxalines **22, (D)** isoquinolines **25** and **(E)** quinolines **30** using Cu-catalyzed MCR.

Zhou et al. synthesized substituted pyridines via a Cu-catalyzed three-component reaction of alkynes **13**, 2-[(amino)methylene]malononitriles **12** and sulfonyl azides **14** (Figure 2A). During the screening of the reaction conditions, the substituents on the pyridines were varied with respect to the solvent. When all three substrates were treated with the Cu catalyst in the presence of triethylamine (TEA) **16**, an anionic intermediate **D** was formed. DMF at 50°C under N_2_ afforded 6-amino-2-iminopyridine **18** by nucleophilic vinylic substitution (S_N_V), followed by intramolecular cyclization of an anionic intermediate **D**. THF at 20°C–25°C afforded 4-amino-2-iminopyridine **17** via direct intramolecular cyclization of the anionic intermediate **D** (Figure 2B) (Zhou et al., 2013).

Gers et al. also reported that the multicomponent reacts with the terminal alkyne **13** to produce substituted quinoxalines **22** (Figure 2C). The electron rich π-nucleophiles were reacted in presence of oxylchloride **20,** thereby furnishing ynedione **23**; this step is called gloxylation. Ynedione **23** further underwent Stephens−Castro coupling with 1,2-diaminoarenes **21**, a cyclocondensation reaction that resulted in the formation of 2-substituted 3-ethynyl quinoxalines **22**. The high fluorescence of the synthesized compounds renders them ideal sensors for protein interactions (Gers et al., 2014).

Ramanathan and Pitchumani reported a Cu-catalyzed MCR between sulfonyl azide **13** and terminal alkyne **13** (Figure 2D). The expected pyrimido [1,6-a]quinolines **25** were prepared using a tandem technique involving the ketenimine-based [3 + 2]/[2 + 2 + 2] intermolecular cycloaddition of alkyne **20**, sulfonyl azide **14**, and quinoline **24**. In this simple, atom- and step-efficacious procedure, copper (I) promotes the cycloaddition of azide-alkyne, followed by ring-rearrangement/ketenimine formation/regio- and stereoselective termolecular cycloaddition and dehydrogenation cascade, to selectively yield pyrimido [1,6-a]quinoline *E*-isomer (Ramanathan and Pitchumani, 2015).

Mondal et al. synthesized quinolones **30** using a Cu catalyst by coupling various anilines **26**, terminal alkynes **13** and aldehydes **27** under solvent-free conditions (Figure 2E). The expected 4-substituted, 2,4-disubstituted, and thermally labile sugar-based chiral quinolines **30** were obtained in good yields via cyclization of the C≡C bond. Terminal alkyne **13** was activated when treated with a Cu catalyst, which further formed the propargyl amine **31** intermediate through the formation of C−C and C−N bonds between aldehyde **27** and aromatic amine **26**. The propargylamine **31** intermediate can be used as a Cu^I^ procatalyst, which initiates sp^2^ C–H activation with cyclization involving transient Cu^III^ species. The reductive elimination step helps obtain quinolones and the Cu^I^ catalyst (Mondal et al., 2016).

**FIGURE 3:**
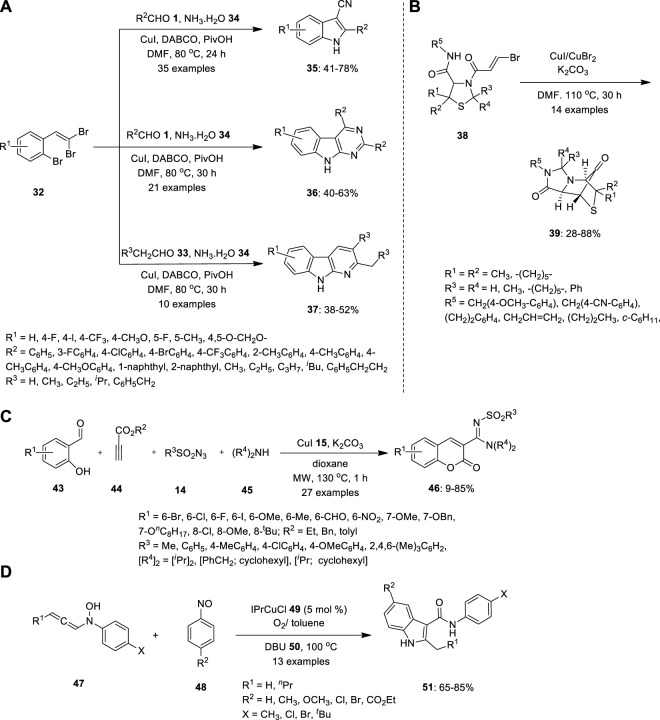
Copper catalyzed MCR for synthesis of **(A)** indoles **35**, pyrmidoindles **36** and and pyridoindoles **37**, **(B)** bridged N- heterocyclic compound **37**, **(C)** 3N-sulfonylamidine and **(D)** functionalized indoles **51**.

Li et al. provided a selective methodology for the synthesis of indole and its derivatives throughout copper-catalyzed one-pot multicomponent cascade reactions. 1-bromo-2-(2,2- dibromovinyl) benzenes **32** were treated with aldehydes **1** and aqueous ammonia **34** in the presence of Cu catalyst in DMF at 80°C (Figure 3A). The ligands 1,4-diazabicyclo [2.2.2]octane (DABCO) and pivalic acid (PivOH) were used as additives to facilitate Cu catalysis. The resulting products also differed depending on the reaction time and substrate ratio. When the reaction was carried out in a 1:1 M ratio of aqueous ammonia to DMF for 24 h (condition A), 3-cyanoindoles **35** was formed. Whereas when the reaction was performed with a 0.5:3 M ratio of aqueous ammonia to DMF for 30 h (condition B), the produced sequence of 9H-pyrimido[4,5-b]indoles **36** disregarded the steric and electronic nature of the substituents. Similarly, pyrido[2,3-b]indole derivatives **37** were obtained under condition B using aliphatic aldehydes **33** were employed (Li et al., 2015).

(Kroger et al., 2015) investigated the diastereoselective synthesis of a tricyclically annulated and bridged heterocyclic system reinforced by Cu-mediated rearrangement based on bisamides with a thiazolidine substructure. (Figure 3B). Precursors **38** were easily synthesized by a two-step synthetic dispatch known as the Asinger and Ugi reaction, using the concept of consecutive MCRs with 3-thiazolines **40**, trans-bromoacrylic acid **41** and isocyanide **42**. Precursor **38** further reacted with CuI/CuBr_2_ in the presence of K_2_CO_3_ in DMF at 110°C. Consequently, product **39** was obtained by intramolecular ring-closing reaction between the secondary amide and brominated alkene in moderate yield (Kroger et al., 2015).

Propiolate **44**, salicylaldehyde **43**, sulfonyl azide **14**, and secondary amine **45** were coupled to form a four-component tandem copper-catalyzed 3-*N*-sulfonylamidine coumarin **47** (Figure 3C). Using this one-pot procedure, Murugavel et al. obtained functionalized coumarin structural frameworks in moderate-to-high yields. Thus, the cycloaddition of **44** with **14** may produce ketenimine. Nucleophilic addition of the amine **45** to the ketenimine may furnish amidine that can react with the aldehyde **44** to give cationic intermediate, which may cyclize to produce **46** by transesterification. When the reaction was conducted at room temperature, 2-imino esters and substituted phenols were formed as byproducts, which decreased the yield of the expected coumarin. The reactions were performed under microwave irradiation. Surprisingly, the expected coumarin **46** was formed as the major product, essentially dominating other by-products (Murugavel, and Punniyamurthy, 2015).

Because the reaction entails a notable structural rearrangement, the 1,8-Diazabicyclo[5.4.0]undec-7-ene (DBU) **50** -mediated indole synthesis is mechanistically intriguing. In 2016, Sharma and Liu proposed process, the [3 + 2]-annulation reactions were carried out using nitrosobenzenes **48** and N-hydroxyaniline **47** in the presence of an IPrCuCl **49** additive and O_2_ in cold toluene (Figure 3D). The resulting isoxazolidin-5-ol derivatives were heated in (DBU) **50** in toluene. DBU can help the initial ketal’s ring open to generate intermediate, which then catalyses the ring’s rearrangement and leads the formation of ketenimine isomer, which is then converted as the O−linkage isomer. In contrast to its N−linkage isomer, O−linkage isomer is chemically reactive to undergo a 3,3-sigmatropic shift, resulting in the 3-oxo-2-arylamides and the desired product **51** with moderate to good yield (Sharma, and Liu, 2016).

**FIGURE 4:**
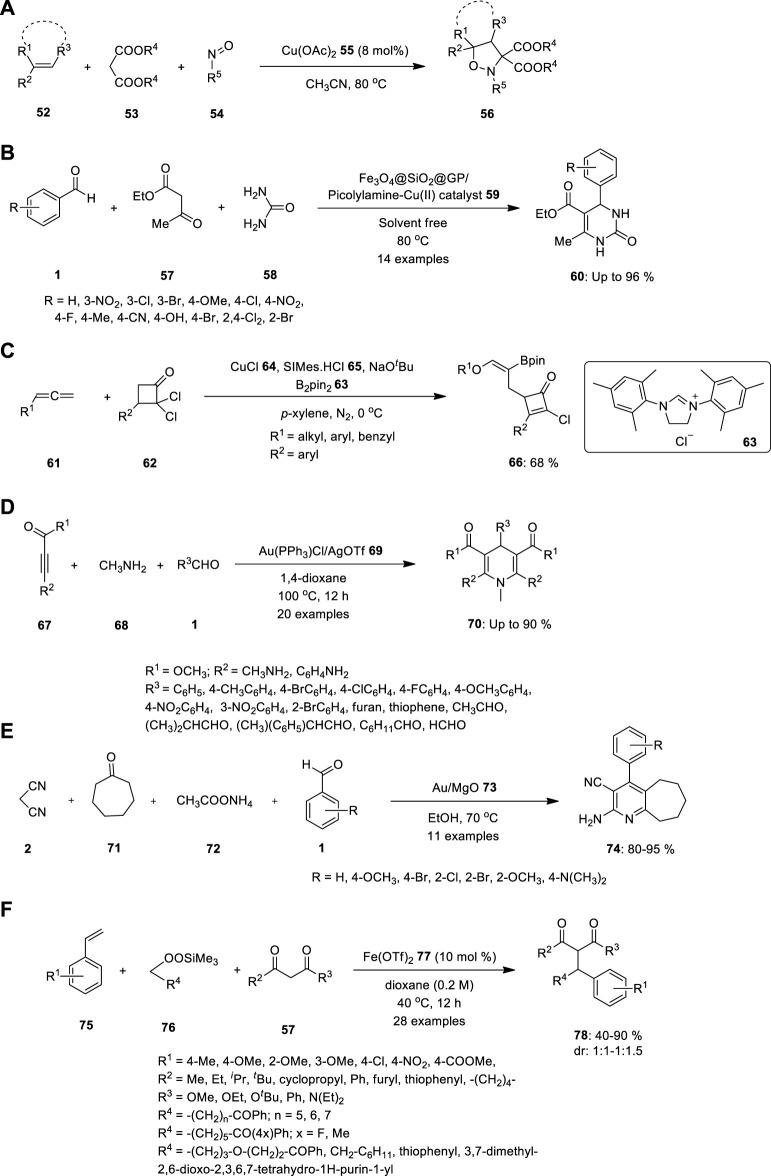
Synthesis of **(A)** *N,O*—heterocyclic compound **56**, **(B)** 3,4-dihydropyrimidinones **60, (C)** polysubstituted cyclobutenone **66** using copper catalyzed MCR, **(D)** N-substituted dihydropyridines **70, (E)** multisubstituted pyridine compounds **74** using Au-catalyzed multicomponent reaction, **(F)** Dicarbofunctionalization of vinylarenes **75** in presence of catalytic Fe(OTf)_2_ **77**.

Malonate **53**, nitrosoarene **54**, and alkene **52** undergo one-pot three-component cycloaddition under the influence of Cu(OAc)_2_ **55** (Figure 4A). Using this technique, Wu et al., 2023 produced various isoxazolidines in good to outstanding yields. Further, they conducted mechanistic studies, which revealed that the simple production of nitrone intermediates via the Cu(OAc)_2_-catalyzed reaction of malonates with nitrosoarenes is an essential step in this catalytic system (Wu et al., 2023).

Rezayati and team reported biginelli annulation reaction catalysed by heterogeneous magnetic silica-coated picolylamine copper complex to synthesis of biologically active 3,4-dihydropyrimidinones **60**. The catalyst Fe_3_O_4_@SiO_2_@GP/Picolylamine-Cu (II) **59** was synthesized easily using chemical attachment of the picolylamine compound on Fe_3_O_4_@SiO_2_@GP, followed by treatment with copper salt in ethanol under reflux conditions. The resulting catalyst system was successfully used in the Biginelli reaction through a variety of compounds such as aromatic aldehyde **1**, urea **58**, and ethyl acetoacetate **57** under solvent-free conditions or ethylene glycol at 80 °C and yielded the desired products with high conversions with powerful reusability (Figure 4B). The current approach was convenient and clean, and only 0.01 g of the catalyst could be used to perform the reaction. Also, the Fe_3_O_4_@SiO_2_@GP/Picolylamine-Cu (II) nanocatalyst **59** could be recycled by an external magnet for eight runs with only a significant loss in the product yields (Rezayati et al., 2022).

In addition, the researchers inspired by the selectivity in multicomponent reactions demonstrated by metal catalysts. To prove that, Liu and team reported copper catalyzed regio-and stereoselective three-component coupling of allenyl ethers with gem-dichlorocyclobutenones **63** and bis (pinacolato) diboron **62**. The reaction yielded a variety of highly functionalized cyclobutenone products **67** tethering with an alkenylborate fragment (Figure 4C). The polysubstituted cyclobutenones also underwent diverse transformations (Liu et al., 2023).

## 4 Gold

Wang S. et al. described the one-pot MCRs of activated alkynes **67**, methanamine **68**, and aldehydes **1** catalysed by gold to the synthesis of bioactive N-substituted dihydropyridines (DPH) **70**. The reaction provided a convergent strategy for generating a C–C bond and a C–N bond for the synthesis of N-substituted DHP derivatives in moderate to high yields (Figure 4D). Whether an electron-withdrawing or electron-donating group was introduced on the phenyl ring, the reaction proceeded without mishap and produced the corresponding N-substituted DHPs in good yields (Wang et al., 2013).

Pagadala R et al. demonstrated Au/MgO **73** to be a highly effective and reusable catalyst for multicomponent coupling reactions at 70°C (Figure 4E). The synthesis of multisubstituted pyridines **74** resulted in high yields and rapid reaction rates. Reaction proceeded through the Knoevenagel condensation of aldehyde **1** with sp^3^ carbon of malononitrile **2**, double bond further reduced by Michael addition, which further underwent cyclization with the help of ammonium acetate **72**. After a completion of reaction, the novel catalyst can be easily recovered and re-utilized without any loss of catalytic activity, as a result of its simple processing (Pagadala et al., 2014).

## 5 Iron

Recently, Xu et al. introduced a novel strategy for developing a set of carbon–carbon bonds using styryls **75** alongside 1,3 dicarbonyl substrates **57**, catalyzed by the existence of catalytic Fe(OTf)_2_ **77** under optimized reaction conditions (Figure 4F). Vinylarenes with various substituents, along with numerous alkylsilyl peroxides **76** and β-keto carbonyl substrates **57**, can be employed in this process. Using the same reaction conditions, a novel benzopyran derivative **78** was created from 2-hydroxystyrene, which is an important molecule with various biological characteristics (Xu et al., 2022).

## 6 Iridium

**FIGURE 5:**
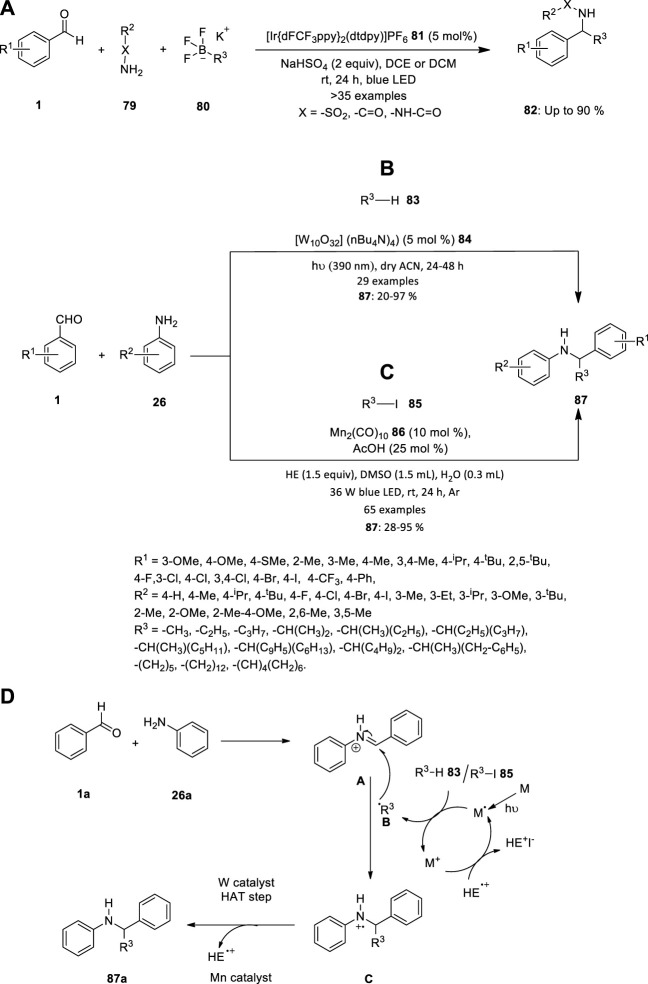
**(A)** Iridium catalyzed photoredox Petasis reaction, Synthesis of secondary amines using **(B)** TBADT **84** and **(C)** Mn_2_(CO)_10_ **86** catalyst, **(D)** Proposed mechanism for the synthesis of secondary amine **89**.

Vytla et al. reported an efficient three-component photoredox-catalyzed Petasis reaction. Under the optimized conditions using {Ir[dFCF_3_PPy]_2_(dtbpy)}PF_6_ **81** as the photocatalyst, a wide range of sulfonamides, amides and hydrazides can efficiently undergo imine formation with various benzaldehydes **1** followed by radical α-alkylation with alkyltrifluoroborates **80** in presence of sodium hydrogen sulfate (Figure 5A). The operationally simple protocol allows rapid access to structurally diverse α-substituted secondary sulfonamides, amides and hydrazides **82** in moderate to high yields (Vytla et al., 2022).

## 7 Manganese and tungsten

Due to the prevalence of secondary amines in agrochemicals, drugs, natural products, and small-molecule biological probes, there have been efforts to accelerate the synthesis of molecules with an amine functional group. The two different metal catalysts were introduced to synthesis the secondary amines in 2021. Due to the benefits that the direct functionalization of these potent C-H bonds holds, the prevalence of non-activated aliphatic C-H bonds has been the focus of ongoing research. It is clear that this discipline has advanced thanks to the discovery of photoredox catalysis, which offers a variety of solutions to problems caused by the inertness of unactivated C-H bonds. Pillitteri et al. investigated the production of C-sp^3^ centred radicals from aliphatic C-H bonds (Figure 5B). This is made simple by the use of polyoxometalate tetrabutylammonium decatungstate (TBADT) **84**. Despite the widespread use of this photocatalyst, only a small number of radical acceptors have been used, and there aren't many reports on multicomponent reactions, primarily because of polarity match and chemoselectivity problems. Therefore, the team achieved a generally applicable method for the mild reaction conditions alkylation of *in situ* generated imines to produce functionalized secondary amines **87**. The innate electrophilicity of imines towards radical addition is exploited in this atom-economic transition, and the starting materials' electronic characteristics are tuned to produce a wide range of products (Pillitteri et al., 2021).

Wang et al. described the three-component alkylation reactions of imines (produced *in situ* by condensation of benzaldehydes **1** and anilines **26**) with unactivated alkyl iodides **85**, catalysed by inexpensive and widely available Mn_2_(CO)_10_ **86**, are here as a mild, operationally straightforward method for the synthesis of secondary amines **87** (Figure 5C). This procedure is a versatile, adaptable method for the synthesis of secondary amines **87** because it is compatible with a variety of sensitive functional groups and doesn’t call for a significant amount of the alkylating reagent (Wang et al., 2021).

The mechanism depicted in Figure 5D is what causes the amination reactions to happen. When Mn catalyst is exposed to the blue LED, metal radical is produced. This metal radical takes iodine/hydrogen from R^3^−I **87** to produce the nucleophilic radical species **B** and metal cation. Radical **B** combines with iminium ion **A** (which was created *in-situ* by a condensation process between **26a** and **27a** and then protonated by AcOH) to create radical cation **C**. After further reducing **C**, a tertiary amine product **89** is produced, and this produces a radical cation called HE^+^, which combines with metal cation to regenerate the active catalyst radical and end the photocatalytic cycle. Whereas, while using tungsten compound used as catalyst, R^3^−H **85** produced radical **B** and hydrogen radical. Radical **B** combines with iminium ion **A** to create a radical **C**, which underwent hydrogen atom transfer (HAT) step to produce a tertiary amine product **89**.

## 8 Nickel

**FIGURE 6:**
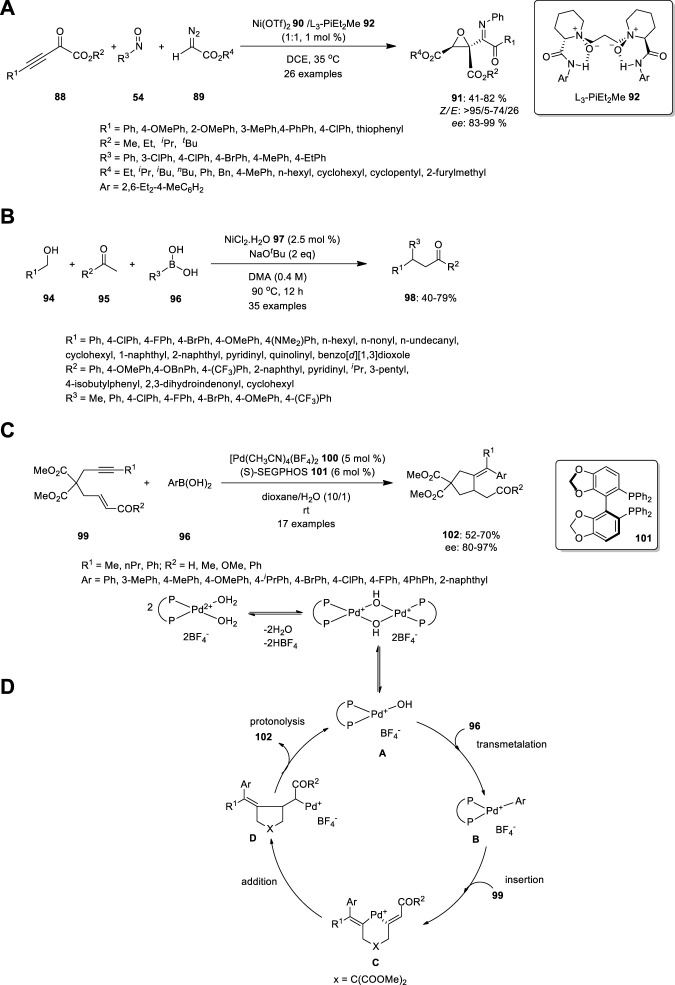
Synthesis of **(A)** imino ketone-substituted multifunctional chiral epoxides, **(B)** β,β-disubstituted ketones **98** using Ni complex, **(C)** An enantioselective arylative cyclization of alkyne-tethered enals or enones catalyzed by cationic Pd(II) and **(D)** proposed mechanism.

Zhang et al. used chiral N,N′-dioxide/Ni(OTf)_2_ complex **90** to develop an enantioselective three-component reaction of 2-oxo-3-ynoates **88**, nitrosoarenes **54** and diazoacetates **89** (Figure 6A). This process in mild reaction conditions produces imino ketone-substituted multifunctional chiral epoxides **91** in ordinary to good yields (up to 82%), outstanding diastereo- and enantioselectivities (99% *ee* and up to >95/5 *dr*), and high *Z/E* ratios (up to >95/5 *Z/E*). Essentially, the imino group of product **91** is hydrolyzed at a low pH (<7) to afford the corresponding ketone. Zhang et al. carried out the same reaction in different orders, including nitrosoarenes **54** and diazoacetates **89**, thereby producing nitrone. Subsequently, the nitrone was treated with 2-oxo-3-ynoates **88** to produce aziridine **93**, which is not hydrolyzed (Zhang et al., 2020).

Recently, Balakrishnan et al. treated alcohols **94** and ketones **95** with boronic acid **96** in the presence of NiCl_2_ **97** to produce β,β-disubstituted ketones **98** (Figure 6B). This can be achieved using hydrogen borrowing strategy, which is a cost-effective technique for the α–functionalization of ketones. The alcohol **94** reduced as aldehyde by Ni catalyst, which further underwent aldol condensation reaction with aryl methyl ketone **95** to create enone. Addition of Ni-Ph catalyst to the enone generate 1,4 addition, which further produce the target molecule **98**. This methodology is tolerated by inactivated alkyl alcohols, long-alkyl chain alcohols, and sterically crowded alcohols. Methyl-substituted boronic acids reduced the yield after observing the effect of the substrate in boronic acid. Moreover, the coordination of the oxygen and sulfur atoms to the nickel center rendered thiophene and furan entirely inert (Balakrishnan et al., 2022).

## 9 Palladium

Shen et al. developed an enantioselective arylative cyclization of alkyne-tethered enals or enones catalyzed by cationic Pd(II) via the carbopalladation of alkynes (Figure 6C). The acyclic allylic 2-alkynoate **99** was treated with aryl boronic acid **96** in the presence of [Pd(dppp)(H_2_O)_2_](BF_4_)_2_ **103** in toluene/H_2_O. The expected product, (E)-2-(5-oxo-4-(1-phenylethylidene)tetrahydrofuran-3-yl) acetaldehyde **104**, was obtained in 41% yield along with the uncyclized product, (*Z*)-4-oxobut-2-en-1-yl (*E*)-3-phenylbut-2-enoate **105** (48%). Subsequently, they studied the asymmetric arylative cyclization by introducing a chiral ligand to increase the yield and enantioselectivity. High enantioselectivities were observed when biphenyl or binaphthyl motifs **101** were present in the bisphosphine ligands, thereby resulting in high enantioselectivities with moderate yields, probably owing to the fierce conjugate addition side reactions. The reaction was performed in the absence of water and the yield decreased with increasing temperature (Shen et al., 2012).

The Pd hydroxo complex **A** is the active catalytic species, while arylboronic acids **96** form arylpalladium species **B** through transmetalation. Carbopalladation yields vinylpalladium intermediate **C**, followed by carbon-carbon double bond insertion, resulting in intermediate D. The protonolysis of the C-Pd bond generates the product and regenerates Pd(II) species **A**. The Pd(II) catalytic cycle is a plausible mechanism for cationic Pd(II)-catalyzed transformations, with halogen-substituted phenylboronic acids and allylic esters being substrates (Figure 6D). Both aryl-halogen bonds and allylic ester groups are cleaved first in the presence of Pd (0).

**FIGURE 7:**
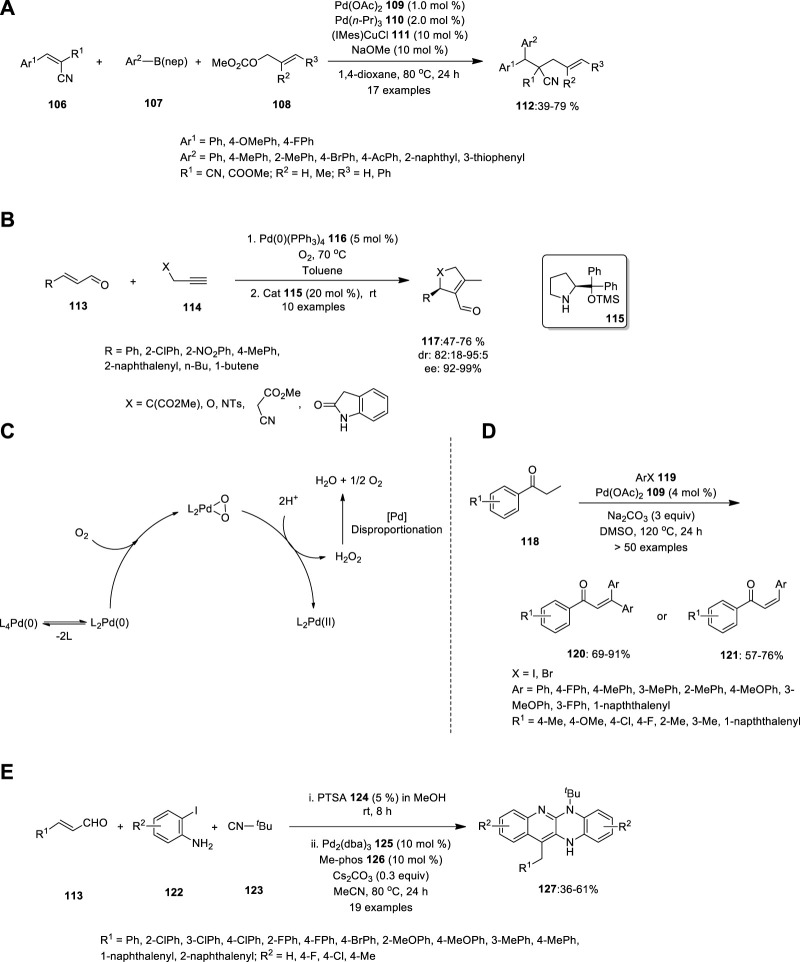
Pd—catalyzed **(A)** carboallylating electron-deficient alkenes **106**, **(B)** intermolecular nucleophilic addition of unmodified aldehydes **113** reactions, **(C)** Oxidation of Pd (0) to Pd(II) by molecular oxygen, **(D)** synthesis of α,β-unsaturated carbonyl compounds **117**, **120,** and **121**, **(E)** Ugi-variant one-pot synthesis of fused tetracyclic quinoline scaffolds **127**.

Using Pd/Cu catalysis, Semba et al. developed a convenient approach for the carboallylation of electron-deficient alkenes (Figure 7A). The yield of the alkenes was unaffected by the presence of electron-withdrawing and electron-donating groups at the para-position on the benzene ring of **112**. Hence, using widely available alkenes **106**, allylic carbonates **108** and organo-boron compounds **107**, this approach may produce numerous carbon skeletons that are well-tolerated by numerous functional groups, including bromo, cyano, alkoxycarbonyl, and acetyl moieties (Semba et al., 2019).

Another chiral ligand was reported in 2014 by Santoro et al., who investigated the mechanism of the palladium and amine co-catalyzed intermolecular nucleophilic addition of unmodified aldehydes to alkynes (Figure 7B). Based on experimentations and theory, they reported that the C–C bond-forming reaction is presumably a two-step carbocyclization of enaminynes catalyzed by a Pd(II) species. In the first step, the α,β-unsaturated aldehyde **113** reacts with chiral amine **115**, thereby forming a nucleophilic enamine, which further attacks the electrophilic Pd(II). This is followed by insertion into the alkyne **114** bond, which produces a syn carbopalladation intermediate. Moderate isolation yields were obtained with good enantiomeric excess ratios.

Predominantly, the catalytic cycle is connected to complete enantioselective aerobic allylic alcohol oxidation using a Pd complex with a chiral amine. Owing to the presence of Pd^II^ (PPh_3_)_2_O_2_ in the catalytic cycle, no other oxidizing agent is required to convert Pd(0) to Pd(II) (Figure 7C). Hence, “Pd oxidase catalysis” and “metal/organo cooperative catalysis” can be used in environmentally friendly and sustainable chemistry (Santoro et al., 2014).

Gandeepan et al. synthesized an extremely substituted α,β-unsaturated carbonyl species using a novel Pd catalyst (Figure 7D). Unlike the Heck-type coupling reaction, this reaction can produce an effective yield with low catalyst loading and desist other lavish metal oxidants or ligands. Saturated ketones, aldehydes, and esters were treated with aryl halides under optimized reaction conditions to afford α,β-unsaturated carbonyl compounds in good to excellent yields. When 2.5 equivalents of aryl iodide were employed in the reaction, monosubstituted α,β -unsaturated compounds **121** were produced; however, when 3.5 equivalents of aryl iodide were used, disubstituted α,β -unsaturated compounds **120** were formed as the major product (Gandeepan et al., 2014).

In the same year, Che et al. carried out the Ugi-variant MCR and Pd-catalyzed one-pot synthesis of bis-annulation with substrates **113**, **122** and **123,** and PTSA **124** in MeOH for 8 h at room temperature. The crude product was concentrated, followed by the addition of Pd_2_(dba)_3_ **125**, Me-phos **(126** and Cs_2_CO_3_ in MeCN at 80°C for 24 h (Figure 7E). The fused tetracyclic quinoline scaffolds **127** were obtained in moderate-to-good yields. The electronic properties of the substituents on the aromatic ring of the aldehyde did not influence the reaction rate, whereas anilines with either electron-donating or electron-withdrawing groups demonstrated reactivity (Che et al., 2014).

Wang et al. reported the synthesis of chiral 3,3-disubstituted 3,4-dihydroisoquinoline and tetrahydroisoquinoline derivatives, which are important building blocks in organic synthesis for biological evaluation and ligand designing. Essentially, they carried out Pd-catalyzed enantioselective imidoylative C (sp^2^)−H bond activation reaction by desymmetrization of dibenzyl isocyanide **128** (Figure 9A). Dibenzyl isocyanide **128** was treated with aryl iodine **129** in the presence of the SPINOL-derived phosphoramidite ligand **130** (L*). 3,4-Dihydroisoquinolines **131** bearing a chiral quaternary carbon center were formed in high yields with good enantioselectivities. Further, the reactions were well tolerated with electronically diverse substituents at the ortho- or para-position of aryl iodides, heteroaromatic iodides, bulky groups (such as naphthalene), and substrates bearing two substituents on the phenyl ring of isocyanides (Wang et al., 2017).

In 2014, Zhu et al. synthesized symmetrical and unsymmetrical methylidenefluorene derivatives via a Pd/Cu-catalyzed MCR (Figure 9B). They developed a three-component reaction combining cyclic diphenyleneiodonium **132**, alkynes **13**, and boronic acid **96** to produce methylidenefluorenes **133** in a single step. The effect of the substituents was examined, and the strong electron deficiency of the substituted groups on phenylboronic acids and aryl alkynes produced the product in lower yields. By contrast, phenylboronic acids with electron-donating or electron-withdrawing substituents, terminal aryl groups, and alkyl alkynes were well tolerated under the optimized reaction conditions. Hence, numerous unsymmetrical methylidenefluorenes could be synthesized in isomeric form. The steric substituent had no influence on chemoselectivity; by contrast, the electronic effect had a significant influence on chemoselectivity. Electronically poorly substituted unsymmetrical cyclic diphenyliodoniums exhibited high chemoselectivity and *vice versa* (Zhu et al., 2014).

Peng et al. attempted the Pd-catalyzed MCR of propargylic carbonates **134** with isocyanides **135** (Figure 9C). They treated the propargylic carbonate **134** with isocyanide **135** in the presence of Pd(PPh_3_)_2_Cl_2_ **136**, and CsF in DMSO. Surprisingly, two different types of useful N-heterocyclic compounds, **137** and **138**, were produced via the orderly insertion of isocyanides. According to a systematic investigation of the reaction conditions, the temperature and ligands can influence the selectivity for the formation of N-heterocyclic products. When they completed the reaction at room temperature, (*Z*)-6-imino-4,6-dihydro-1H-furo[3,4-b]pyrrol-2-amines **137** was predominantly produced, whereas when 1,1′-Bis (diphenylphosphino) ferrocene (dppf) was employed instead of PPh_3_ at 110°C, (*E*)-5-iminopyrrolones **138** was predominantly produced. Their study provides evidence for the migratory insertion of isocyanides into aryl- and alkenylpalladium species.

**FIGURE 8:**
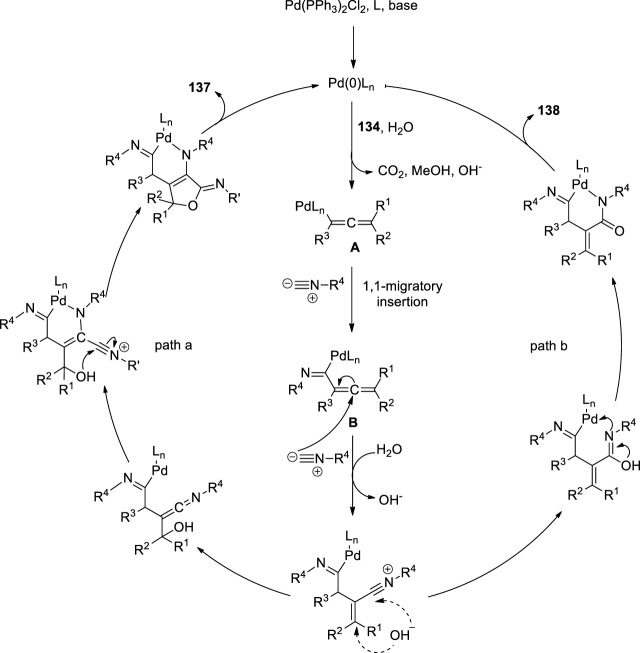
Proposed mechanism.

**FIGURE 9:**
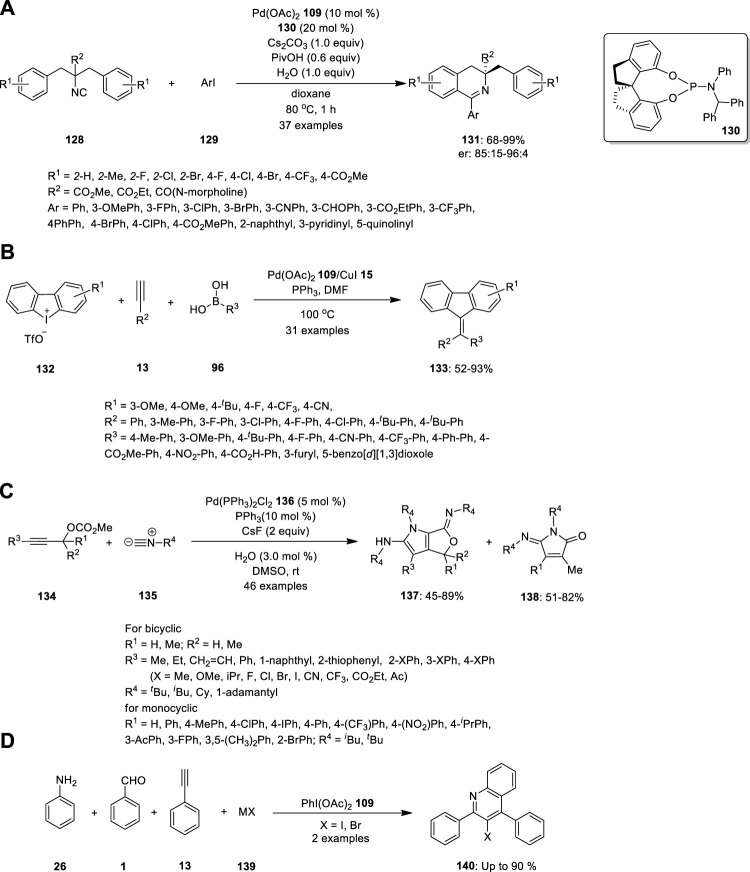
Pd-catalyzed synthesis of **(A)** chiral 3,3-disubstituted 3,4-dihydroisoquinoline **131**, **(B)** methylidenefluorenes **133, (C)** N-heterocyclic compounds **137** and **138, (D)** functionalized 3-haloquinolines **140**.

Proposed mechanism is given in Figure 9. According the mechanism, the reaction initiated with oxidative addition of **134** to the palladium (0) catalyst, producing the allenylpalladium **A**, The in situ-formed allenylpalladium species bearing multiple reaction sites allowed the successive insertion of multiple isocyanides in an orderly manner to become the crucial intermediate **B**. Further, the intermediate **B** has two possible sites C2 and C4 to get attack by H_2_O as a nucleophilic reagent, which determined the selectivity in the synthesis of products **138** and **137** respectively. This mechanism made sense given that compounds **137** were the kinetically preferred products and that they could be produced at room temperature (Figure 8). The thermodynamically preferred pathway for the more stable amide intermediate B, which was different from the keteniminium intermediate III, was path b (Peng et al., 2016).

Lu and coworkers developed by PhI(OAc)_2_-mediated four-component tandem addition/cyclization/oxidation/aromatization process using simple aromatic amine **26**, aldehyde **1**, alkyne **13** and halide salt **139** as a precursors for the exquisite construction of functionalized 3-haloquinolines **140** (Figure 9D). The proposed methodology merging A3-coupling and redox reaction of halide salt, namely, A3-X type tandem reaction, is step economic, functional groups flexible, and potentially applicable to complex molecules under very mild condition (Lu et al., 2022).

## 10 Rhodium

**FIGURE 10:**
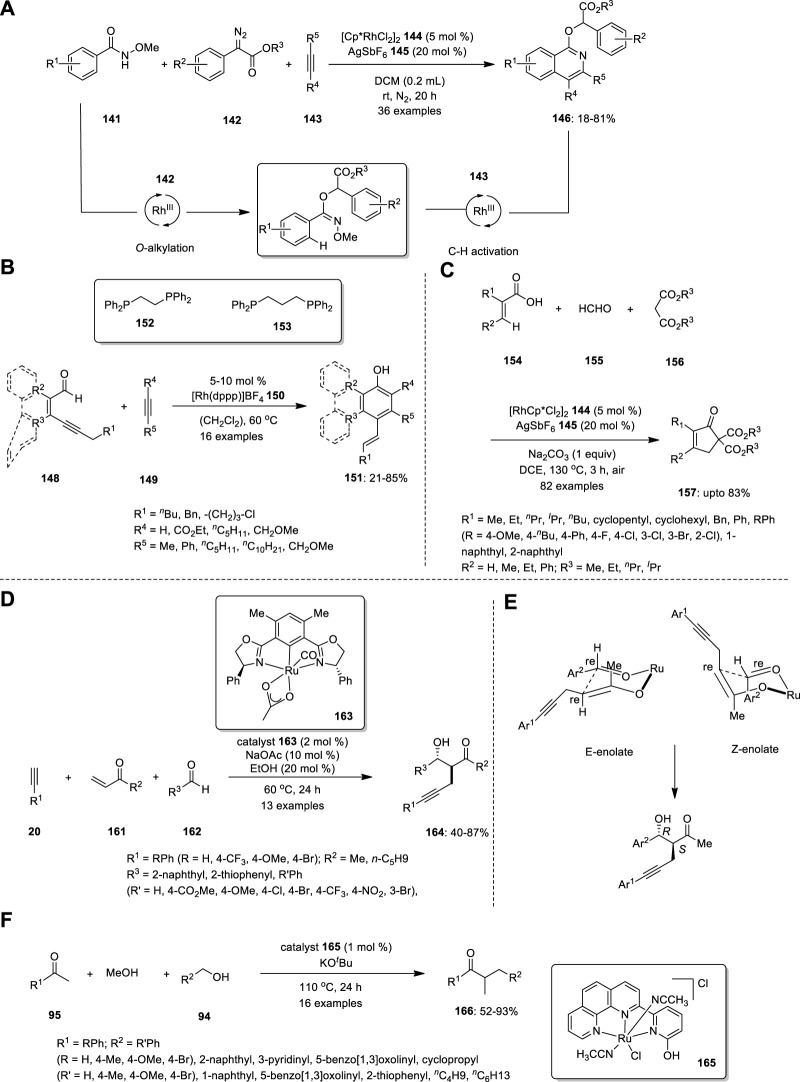
Rhodium (III)-catalyzed synthesis of **(A)** isoquinoline derivatives **146**, **(B)** alcoholic derivatives **151**, **(C)** cyclopentenones **157** and Ru—catalyzed synthesis of **(D)** β−hydroxyketone compounds **164**, **(E)** Isomers of six-membered cyclic transition state, **(F)** α -methylated ketones **166**.

In 2019, Zhou et al. developed a novel strategy for the synthesis of isoquinoline derivative **146** using a rhodium (III)-catalyzed one-pot three-component reaction (Figure 10A). In this reaction, N-methoxybenzamide **141**, α-diazoester **142** and alkyne **143** are smoothly transformed into various isoquinoline derivatives in good yields via successive O-alkylation and inert C–H activation processes in the presence of the Cp*RhIII catalyst at room temperature. When the substrate scope was investigated, both electron-withdrawing and electron-donating groups were tolerated by all substrates, including N-methoxybenzamide **141**, α-diazoester **142,** and alkyne **143**. When asymmetric diarylalkynes were examined, two regioisomeric products were obtained in equal ratios. Subsequently, a reaction sequence was established as a plausible mechanism. N-methoxybenzamide **141** and α-diazoester **142** reacted together to produce oxoimine intermediate **147**, which further combined with diaryl alkyne to produce isoquinoline derivatives **146**. However, an isoquinolone resulting from changing the sequence of treatment of N-methoxybenzamide with diaryl alkyne was not observed (Zhou et al., 2019).

Hojo and Tanaka developed a new methodology for the synthesis of phenol, naphthol, phenanthrenol, and triphenylenol derivatives from readily available conjugated alkynyl aldehydes **148** and alkynes **149** via cationic rhodium(I)/dppp complex catalysis of the aldehyde C−H bond activation/[4 + 2] annulation/aromatization cascade (Figure 10B). The effect of the ligand was examined. Evidently, in the cationic rhodium (I)/dppe complex-catalyzed [4 + 2] annulation, electron-deficient internal alkynes were more reactive, whereas the electron-rich internal alkyne **149** was more reactive when dppf was used as the ligand instead of dppe. All the internal alkynes exhibited high reactivity using a cationic rhodium(I)/dppp complex as a catalyst. The structure of 1,2-Bis(diphenylphosphino) ethane (dppe) **152** and 1,3-Bis(diphenylphosphino) propane (dppp) **153** are shown in Figure 10B. The resulting compound can react to produce pentacyclic compounds via intramolecular Friedel−Crafts alkylation. The synthesis of chrysenol derivatives is also possible by intramolecular olefin metathesis, such as the treatment of the resultant compound with 2nd generation Grubbs catalyst (Hojo, and Tanaka, 2012).

Yu et al. established an efficient Rh-catalyzed protocol for the synthesis of cyclopentenones based on a three-component reaction of acrylic acids **154**, formaldehyde **155**, and malonates **156** via vinylic C−H activation (Figure 10C). An MCR involving the profusion of formaldehyde **155** and malonate **156** produces multisubstituted cyclopentenones **157** via Michael addition. The substrates were reacted in the presence of rhodium catalyst, and 20 mol% AgSbF_6_ **145** afforded the corresponding 5,5- diester cyclopentenone **157**. When various alkyl halides were treated with 5,5- diester cyclopentenone and 1 equiv of Na_2_CO_3_ in MeOH, 5-alkylated cyclopentenones **158** were successfully produced. In addition, the ester group of cyclopentenone could be easily removed by decarboxylation. Moreover, this strategy can produce hexahydro-1H-inden-1-one derivative **159** when cyclohexene-1-carboxylic acid **160** is used instead of acrylic acid **154** (Yu et al., 2021).

## 11 Ruthenium

Ubukata et al. described direct conjugate alkynylation and subsequent aldol reaction-mediated three-component coupling of commercially available alkyne,-unsaturated ketones, and aldehydes (Figure 10D). The advantage of this catalytic system is the commercial accessibility of the substrates. Using this approach, they effectively prepared a range of novel β−hydroxyketone compounds **164** with α−propargyl groups with excellent yields and good chemoselectivities. However, building complex and functionalized organic compounds from accessible starting materials in a one-pot process using this synthetic technique is challenging, although the enantioselectivity is moderate.

The absolute configuration of the main anti-isomer S (α), R (β) could be obtained by C−C bond formation between the *re* face of the aldehyde and the *re* face of the enolate in a six-membered cyclic transition state (Figure 10E). This further confirmed by X-ray diffraction analysis. Control investigations revealed that the Ru−O−enolate species produced by conjugating an alkyne to enone functions as a crucial intermediary for the subsequent aldol coupling process (Ubukata et al., 2016).

For the first time, ketones, alcohols, and methanol were successfully coupled via cooperative Ru(II)-catalyzed tandem three-component coupling (Figure 10F). Surprisingly, under mild reaction conditions, numerous functionalized acetophenones, benzyl alcohol derivatives, and methanol were successfully linked in tandem to produce the corresponding α-methylated ketones **139**. The regioselective conversion of the final ketone products to acetanilide derivatives increased the synthetic usefulness of the reaction. The catalyst loading was decreased to 0.5 mol% at 85°C for the methylation of ketones using methanol as a methylating agent, and it was further decreased to 0.1 mol% at 120°C. To understand the catalytic cycle and the higher reactivity of the bifunctional catalyst, numerous kinetic experiments and DFT calculations were conducted. This tandem three-component coupling procedure is particularly appealing because of the straightforward use of air- and moisture-stable, nonphosphine-based Ru(II) catalysts (Chakrabarti et al., 2017).

## 12 Conclusion

Complex organic compounds are typically synthesized via multistep processes, which typically consume more time, energy, and solvent. Instead, an appropriate alternative to multistep synthesis is MCR, which is more efficient, and time- and energy-saving compared with multistep synthesis. However, MCR has a drawback in regard with its byproducts and selectivity. Thus, metallic catalysts have been introduced to enhance its selectivity. Essentially, the maximum amount of starting materials is consumed when metal catalysts are used, which limits the amount of byproducts. In this study, we summarized the current available literature on metal catalysts utilized in mMCRs for synthesizing organic compounds.
